# Exploring the Nutritional Potential of Microalgae in the Formulation of Bakery Products

**DOI:** 10.3390/foods13010084

**Published:** 2023-12-26

**Authors:** Israel Hernández-López, Maribel Abadias, Virginia Prieto-Santiago, Ángela Chic-Blanco, Jordi Ortiz-Solà, Ingrid Aguiló-Aguayo

**Affiliations:** IRTA, Postharvest Programme, Parc Agrobiotech Lleida. Parc de Gardeny, Edifici Fruitcentre, 25003 Lleida, Spainvirginia.prieto@irta.cat (V.P.-S.); angela.chic@irta.cat (Á.C.-B.); jordi.ortiz@irta.cat (J.O.-S.); isabel.abadias@irta.cat (M.A.)

**Keywords:** microalgae, sensorial acceptance, protein, antioxidant activity

## Abstract

Microalgae have positioned themselves as an innovative and sustainable source of bioactive compounds and high nutritional value. The selection of a suitable food carrier is important to ease its consumption, and to preserve bioactivity through food processing. The aim of this study was to assess the suitability of different microalgae in baked products. Crackers and grissini were produced following a specific formulation, with percentages ranging from 1.5 to 3.5% of flour substituted with *Spirulina*, *Chlorella*, and *Tetraselmis* dry biomass in the formulas. Physico-chemical, nutritional, and sensorial characterization was carried out. The incorporation of microalgae led to increased nutritional values, including antioxidant capacity (AOX), total phenolic content (TPC) and protein content with an amino acids’ identification and quantification. Grissini with *Chlorella* at 3.5% and crackers with *Spirulina* at 1.5% levels, showed a higher overall acceptance within the panelists. For amino acid content, *Spirulina* crackers were shown to be rich in alanine, aspartate, and tryptophan, while *Chlorella* grissini stood out for being particularly rich in isoleucine, leucine, lysine, and valine. Thus, *Spirulina* and *Chlorella* could be a sustainable ingredient to formulate baked goods with an enhanced nutrimental matrix without altering their acceptability to consumers.

## 1. Introduction

Due to their high content of nutrients and health-promoting compounds, microalgae have been evaluated on several occasions as innovative ingredients for the formulation of functional food products. This is because they are rich in high-quality proteins and bioactive molecules with health-promoting properties, such as phycocyanin, lutein or astaxanthin. They have also been shown to have a high polyphenol content and are particularly interesting molecules for their antioxidant, digestive enzyme inhibition and anti-inflammatory properties. Their consumption and production have increased in recent years, with an estimate of 27,000 tons by 2024 [1].

Our current lifestyle, ever-growing population and health and ecological concerns are changing food patterns towards plant-based ones. This is encouraging the food industry to research and find economic and ecologically sustainable novel and cost-effective sources of nutrients, such as proteins, and bioactive compounds, that can easily and rapidly produce ingredients or food products of high nutritional value. Recently, Geyik et al. [2] highlighted that up to 120 countries did not produce enough food to meet the essential needs of their population. Microalgae might potentially aid in health and environmental concerns while being considered economical and efficient sources of nutrients and bioactive compounds [3].

The nutritional and health benefits that are attributed to microalgae are due to their composition and high nutrient content. Not only that, but these bioactive components also present in microalgae, including polyphenols, proteins, polysaccharides and carotenoids, have been shown to have anti-inflammatory, antiviral and antioxidant properties. It has also been shown that the consumption of spirulina promotes exercise and improves performance [3,4,5].

Refs. [4,5] have stated that although environmental conditions may affect biochemical composition, microalgae functional ingredients make them suitable for the design of added-value food products [6]. A high protein content may be the main advantage to research the application of microalgae in the agrifood industry. Values up to 60–65% in dry weight have been found in some species, such as *S. platensis* [6]. Essential amino acids can also be found in microalgae, being the principal sulfur-containing amino acids, such as methionine and cysteine [7]. The interest in the carbohydrate’s composition of microalgae is due to their fiber content. Some species of microalgae, in particular *Chlorella*, present β-glucans, as part of the soluble fraction. These compounds have been reported because of their functionality as immune stimulators, antioxidants, and reducers of blood cholesterol [8].

Moreover, in the nutritional and functional profile of microalgae, lipids play an important role, being a source of monounsaturated and polyunsaturated fatty acids, which confers technological properties, as the emulsifying capacity to the food they are incorporated into [7]. Among fatty acids, omega-3 and omega-6 are reported promoters of health in microalgae, such as *Spirulina* [6]. Microalgae micronutrients are of high interest in the agri-food industry due to their richness and functionality. Among them, antioxidant capacity and subsequent health-promoting properties (including antioxidant activity, anti-inflammatory effects, neuroprotective or cancer prevention) [5] have been attributed to pigments. These compounds are responsible for the vibrant color of microalgae; chlorophyll (green), carotenoids (orange) and phycobilins (red and blue) [8].

In particular, chlorophylls, greenish lipid-soluble pigments found in all algae, and particular in *Chlorella*, *Spirulina* and *Tetraselmis*, are of particular interest because of their direct use as a colorant along with health properties related to cancer prevention [8]. Another micronutrients group that could be interesting in microalgae composition are vitamins, mainly vitamin A, K, E and vitamin B12 [9]. It should be noted that the market advantage of *Spirulina* and *Chlorella* is due to the years that they have already been classified as “GRAS” by the FDA and EFSA [10]. They are completely safe for human consumption, as a food or food supplement. In addition, since 2017, *Tetraselmis* chuii has been authorized by the European Union as safe for commercialization. Therefore, the field of work with this microalga is still wide [11].

It is clear and demonstrable that the composition of microalgae makes them a saleable and beneficial option for those who consume them, whether they are athletes or ordinary people. However, as previously mentioned, consumer acceptance is limited to limited products and/or by-products. With that in mind, the purpose of this study is to investigate the addition of microalgae from the three aforementioned genera to baked goods without these necessarily being bread. In this way, the aim is to try to introduce microalgae to daily consumption in a simpler way.

## 2. Materials and Methods

### 2.1. Products Development and Sensorial Evaluation

#### 2.1.1. Microalgae Biomass and Ingredients

The flour used was wheat flour, with the classification type 55, which is defined by the amount of ash in the flour, in this case 0.55%. This flour is the classic flour used for baking and pastries. Baking powder and flours were kindly provided by Calé-Industria e Comercio LDA (Peniche, Portugal). Both *Spirulina* and *Chlorella* biomass were kindly provided by Allmicroalgae Natural Products S.A. (Pataias, Portugal), while *Tetraselmis* biomass was provided by Necton-Companhia Portuguesa de Culturas Marinhas S.A. (Olhão, Portugal).

#### 2.1.2. Baked Goods Preparation

Crackers and grissini were produced following a specific formulation at the pilot plant facilities of IRTA Fruitcentre (Lleida, Spain). Briefly, ingredients listed in Table 1 and Table 2 were mixed with an AM-700 bread dough mixer (Orbegozo, Murcia, Spain), followed by a particular procedure for each product. Flour substitution levels were evaluated in previous studies [12]. These levels were 1.5%, 2.5% and 3.5%. Products were baked on a Rational Oven (Landsberg am Lech, Germany).

Briefly, dough for crackers was mixed for 4 min at a low speed and 3 min at medium speed, until homogenous consistency was achieved. It was then left to rest for 10 min. Dough was latter flattened into a height of 2 mm and cut into 8 cm diameter circles for crackers. The baking took place at 170 °C for 14 min.

Grissini were mixed for 4 min at a low speed and 3 min at medium speed, until the dough had a homogenous consistency. They were then left to rest under a damp cloth for 10 min. The dough was latter flattened into a height of 2 mm and cut into 7 cm long sticks. These were baked for 18 min at 170 °C. 

#### 2.1.3. Physical Characterization

The moisture content was determined using the AACC Method 44–15.02. Briefly, a small sample of each product (in this case 5 g) was measured with a FV-120 precision balance (Gram Precision S.L, Barcelona, Spain); the sample is placed in a small metal dish and the weight of the sample is noted. A small modification was made in this step since the sample was placed in a small piece of aluminum foil instead. 

This sample was introduced onto a shelf of the oven for 24 h at 100 °C. The sample was removed and cooled in a specific glass container that prevents changes in the moisture of the sample while cooling.

Finally, the sample with its container was reweighed. The results were given as a percentage of moisture and calculated with Equation (1).

Moisture% = (wi − wf)/wi × 100(1)

wi = initial weight of the sample,
wf = final weight of sample.

Three measurements were taken for each formulation and replicated.

The color recordings were taken using a Minolta CR-400 chroma meter (Minolta INC., Tokyo, Japan). CIE values were registered in terms of L* [lightness: black (0)/white (100)], a* [greenness (−60), redness (+60)], and b* [blueness (−60)/yellowness (+60)]. Calibration was carried out using a standard white tile (Y: 92.5, x: 0.3161, y: 0.3321) provided by the manufacturer and the D65 illuminant, which approximates daylight.

The texture profile analysis (TPA) was determined using a TA. XT2 Texture Analyzer (Stable Micro Systems Ltd., Surrey, UK) connected to Exponent software v.5.0.6.0 and equipped with a P/20 aluminum compression probe. The hardness was determined using a knife edge with a slotted insert probe (HDP/BS) as described by Lafarga et al. [12]. Five samples were taken for each formulation and replicated.

### 2.2. Nutritional Characterization, Product Selection and In Vitro Bioavailability Essay

#### 2.2.1. Total Phenolic Content and Antioxidant Activity

The total phenolic content (TPC) was determined by the Folin–Ciocalteu method. The antioxidant capacity was determined using a ferric ion reducing antioxidant power (FRAP) activity assay. The TPC was assessed following the protocol described by Lafarga et al. [13] with some modifications to the extraction process. For the extraction, 3 g of the ground sample was added in centrifuge tubes with 15 mL of methanol (Panreac AppliChem, Llinars del Valles, Spain) at 70% (*v*/*v*) at a sample: methanol ratio of 1:4 (*w*/*v*) and mixed for 20 min at room temperature with a vortex. Samples were then centrifuged using a Sigma 3–18 KS centrifuge (Sigma Laborzentrifugen GmbH, Osterode am Harz, Germany) operating at 14,000 rpm for 10 min at 4 °C. Absorbance was read at 760 nm using a FLUOstar Omega (BMG LabTech, Ortenberg, Germany). 

Antioxidant activity (AOX) was measured using the method: Ferric reducing antioxidant power (FRAP) as previously described by Nicolau-Lapeña et al. [14]. The FRAP reagent was prepared with a mixture of acetate buffer 0.3 M pH 2.6,2,4,6-tris(2-pyridyl)-s-triazine (TPTZ) TPTZ 40 mM in HCl and FeCl_3_·6H_2_O 20 mM in distilled water in a 10:1:1 (*v*:*v*:*v*) proportion. The determination was performed by adding 0.02 mL of the extract to 0.180 mL of the FRAP reagent and incubating it at 37 °C for 20 min in the dark. Absorbance was read at 593 nm using a FLUOstar Omega spectrophotometer (BMG Labtech, Ortenberg, Germany).

#### 2.2.2. Total Protein Content

The total protein content was determined by Lowry’s methodology [15] with some modifications. On a 96-wells microplate were added 10 µL of the sample, 30 µL of NaOH 1 M, 40 µL of distilled H_2_O and 180 µL of Reagent 3. After that, samples were shaken and kept in darkness at room temperature for 10 min. Afterwards, 0.4 mL of reagent 4 was added and kept again in the darkness for 30 min. Finally, absorbance was read at 750 nm using a FLUOstar Omega (BMG LabTech, Ortenberg, Germany).

### 2.3. Sensory Evaluation

Sensory evaluation was undertaken by 39 semi-trained panelists, who were willing to purchase microalgae-containing foods, recruited from the IR-TA Fruitcentre in Lleida, Spain. The sensory evaluation was conducted following the methodology described by Millar et al. [16] with some modifications. Each panelist assessed all the samples and was asked to indicate their opinion on the global acceptance, flavor, firmness and crunchiness using a 9-point hedonic scale (from 1: extreme dislike to 9: extreme like). The acceptability index was calculated as described in previous studies. 

The Sensorial Sciences and Consumers Committee (CCSC) at the Research and Agri-food Technology Institute (IRTA) authorized (CCSC 3/2021) the experimental procedure of the incorporation of microalgae into bakery products and considered that it can be developed with guarantees from the IRTA-Fruitcentre (Parc Agrobiotech. Edifici Fruitcentre, Lleida, Spain), and in accordance with the basic legislation in force on Data Protection (Organic Law 3/2018 and General Regulation EU 2016/679) and the legal requirements linked to the ethical principles on research with human participants (Declaration of Helsinki 1 and the Belmont Report), the measures in place guarantee both data protection and compliance with the ethical principles on research with human participants.

#### 2.3.1. In Vitro Gastrointestinal Essay 

In vitro gastrointestinal digestion was assessed as described by Hernandez-Lopez et al. [17]. Briefly, the method consists of three sequential phases: (i) oral (37 °C, pH 7.0, α-amylase, 2 min), (ii) gastric (37 °C, pH 3.0, pepsin, 2 h) and (iii) intestinal (37 °C, pH 7.0, pancreatin and fresh bile, 2 h). The used pancreatin contained enzymatic components, including trypsin, amylase and lipase, ribonuclease, and protease. A blank sample was prepared using distilled water. TPC and AOX determinations after the intestinal phase were performed in triplicate.

#### 2.3.2. Essential Amino Acids Quantification

For the essential amino acid content (EAA) determination, homogenous samples were extracted following the protocol described by Kıvrak et al. [18] Briefly, 100 mg were weighed into a 2 mL Eppendorf tube and 1 mL of MeOH/H2O (80:20) (*v*/*v*) 0.1% HCOOH was added. The mixture was sonicated for 5 min and subsequently vortexed and then immediately centrifuged at 4 °C at 4000 rpm for 15 min. The supernatant was filtered through a 0.22 µm pore-size PTFE membrane filter. The samples were then spiked into the UPLC equipment with a binary pump and an autosampler for up to 96 vials equipped with refrigeration. 

The UPLC-QToF-MS/MS instrument consisted of a Waters (Milford, MA, USA) Acquity Ultra Performance LC with a Bruker Daltonics (Billerica, MA, USA) QToF-MS, model maXis. Separation was carried out with a Phenomenex (Torrance, CA, USA) C18 column (Luna Omega Polar C18 50 mm × 2.1 mm, 1.6 μm particle size). Some 3 μL of injection volume was used. The column temperature was set at 40 °C. The solvent system consisted of 0.5% aqueous formic acid (Mobile Phase A) and methanol/water (50:50) containing 0.5% formic acid (Mobile Phase B). The chromatographic conditions gradient is specified in Table 3.

### 2.4. Statistical Analysis

Results are expressed by mean ± standard deviation (SD) of three repetitions. The statistical analysis of the experimental data was completed with JMP 13 software (SAS Institute Inc., Cary, NC, USA) using a two-way analysis of variance (ANOVA). The comparison of means was made according to the Tukey test with a significance level of 95% (*p* < 0.05). 

## 3. Results and Discussion

### 3.1. Evaluation of Incorporation of Microalgae in Baked Goods

**Figure 1:**
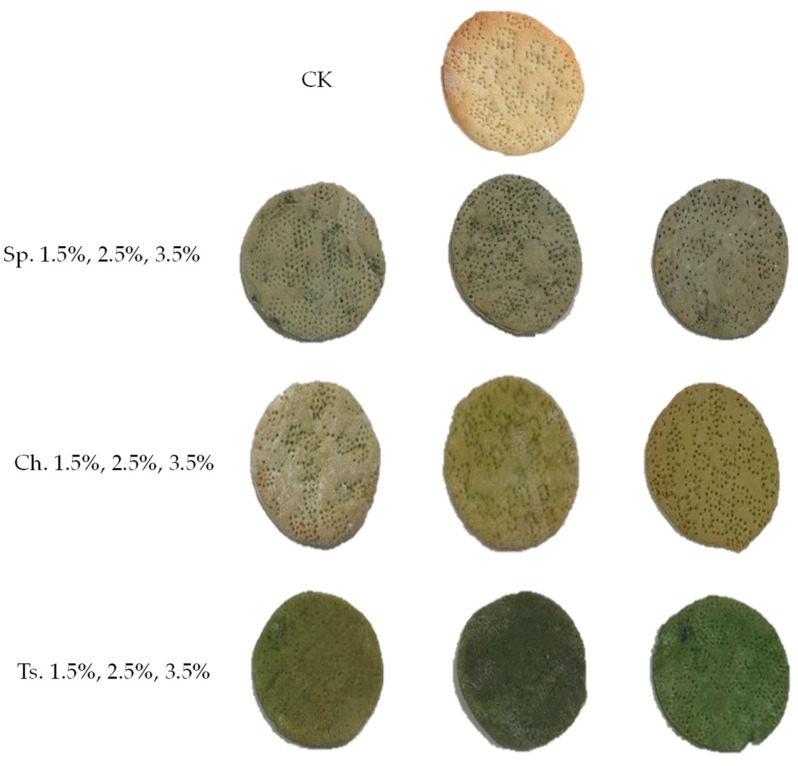
Crackers with microalgae biomass added in three flour substitution levels (1.5, 2.5 and 3.5%). Figure abbreviations Sp. *Spirulina*, Ch. *Chlorella* and Ts. *Tetraselmis*.

**Figure 2:**
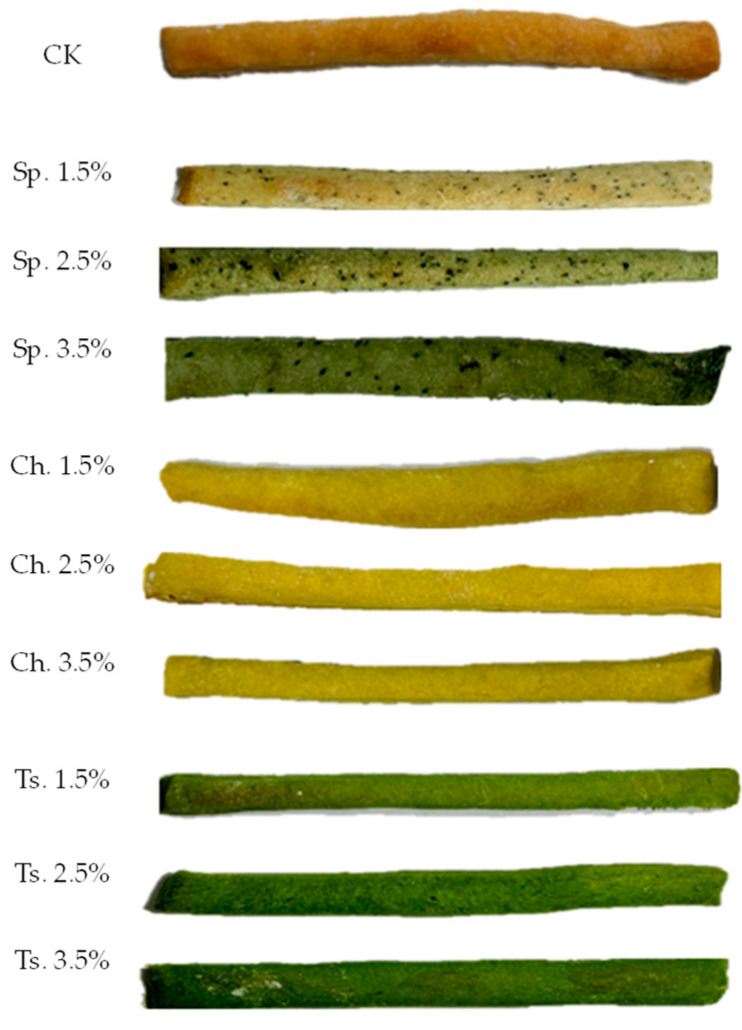
Grissini samples with microalgae biomass added in three flour substitution levels (1.5, 2.5 and 3.5%). Figure abbreviations Sp. *Spirulina*, Ch. *Chlorella* and Ts. *Tetraselmis*.

Microalgae biomass incorporation affected the visual appearance of the grissini and crackers (Figure 1 and Figure 2) when compared to the control (*p* < 0.05). An increase in green coloration was observed as biomass was added for the three strains evaluated (Table 4). The greenish color has been largely accepted before, as reported in Lafarga et al. [19] where they added broccoli co-products into the bread formulation. As the microalgal biomass increased in the formulation, the L* values were lower, evaluating in this way a darker greenish color in the baked products. Both a* and b* values were affected negatively with the increase in the biomass, even though the b* values were higher than the a* values (Table 4).

This behavior was reported previously by Hernandez-Lopez et al. [17], Lafarga et al. [9], and Batista et al. [20] when green microalgae biomass was added to flour-based foods.

**Figure 3:**
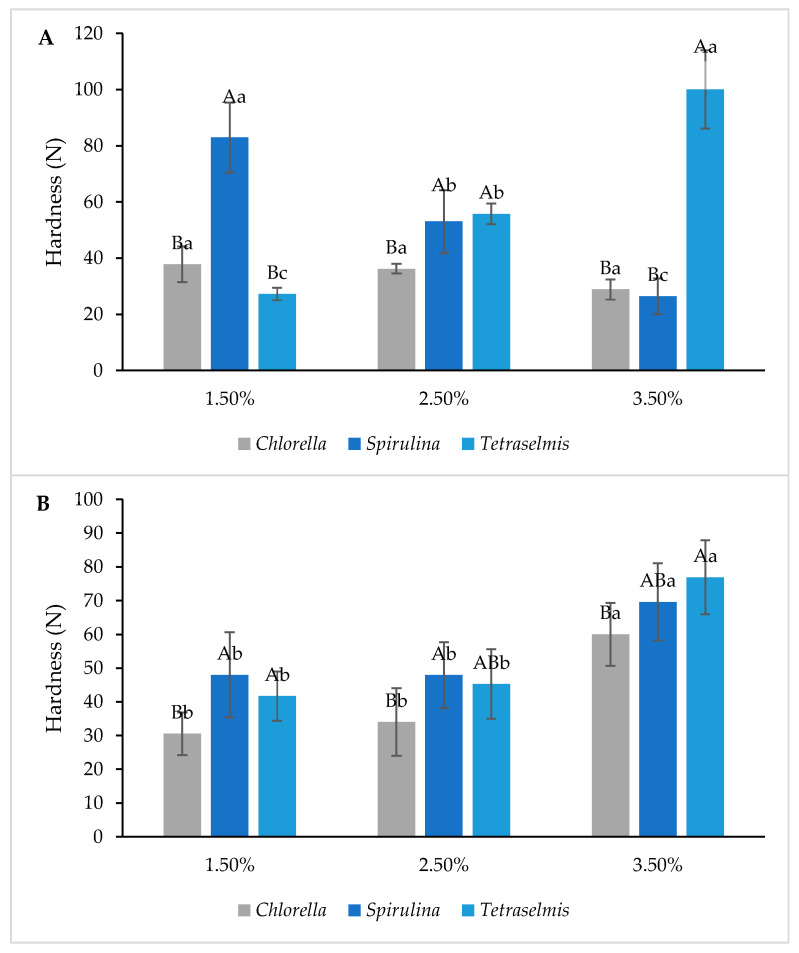
Section (**A**) of the figure represents the textural results of the crackers and section (**B**) is for the grissini results. Different capital letters indicate significant differences between formulations baked with different concentrations with the same microalgae. Different lowercase letters indicate significant differences between microalgae at the same concentration. The criterion for statistical significance was *p* < 0.05.

Textural properties related to the hardness of the formulated products are shown in Figure 3. As observed, the hardness of the crackers decreased while the concentration of Spirulina increased, ranging from 80 N at 1.5% to 26 N at 3.5% concentration. However, no texture differences were observed in this product when adding Chlorella, irrespective of the concentration used. In contrast, crackers containing Tetraselmis exhibited increased hardness with a rising microalgae concentration. This trend was also evident in grissini samples, where higher microalgae concentrations correlated with increased hardness. These results are in line with the ones presented by Batista et al. [20] and differ from those reported by Figueira et al. [21], and Garcia-Segovia et al. [22] where the addition of microalgae to bread with gluten-free rice flour did not present significant changes in the texture. The observed hardness results were related to the moisture content in the samples. The moisture increased significantly in Chlorella grissini samples, ranging from 14 to 18% moisture, compared to the 7% in the CK samples. Crackers produced very different results: when compared to the CK samples, the moisture was lower. The highest result was achieved by Spirulina at a concentration of 1.5%. A decrease in moisture was observed for crackers while the microalgae concentration increased. Cracker results can be compared to those obtained by Lafarga et al. [12] and were higher than those obtained by Batista et al. [20]. A lower moisture content would be preferable in these types of products since they are expected to be dry and crunchy.

### 3.2. Nutritional Quality

**Figure 4:**
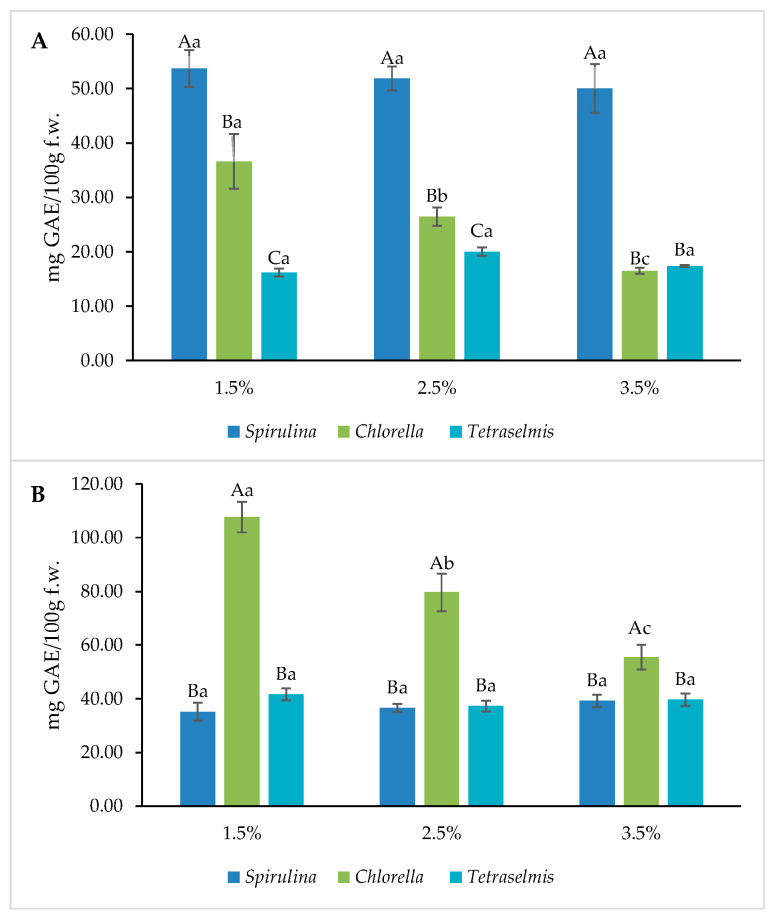
Total phenolic content (TPC) for microalgae containing samples. (**A**): Crackers and (**B**): Grissini. Different capital letters indicate significant differences between formulations baked at different concentrations with the same microalgae. Different lowercase letters indicate significant differences between microalgae at the same concentration. The criterion for statistical significance was *p* < 0.05.

**Figure 5:**
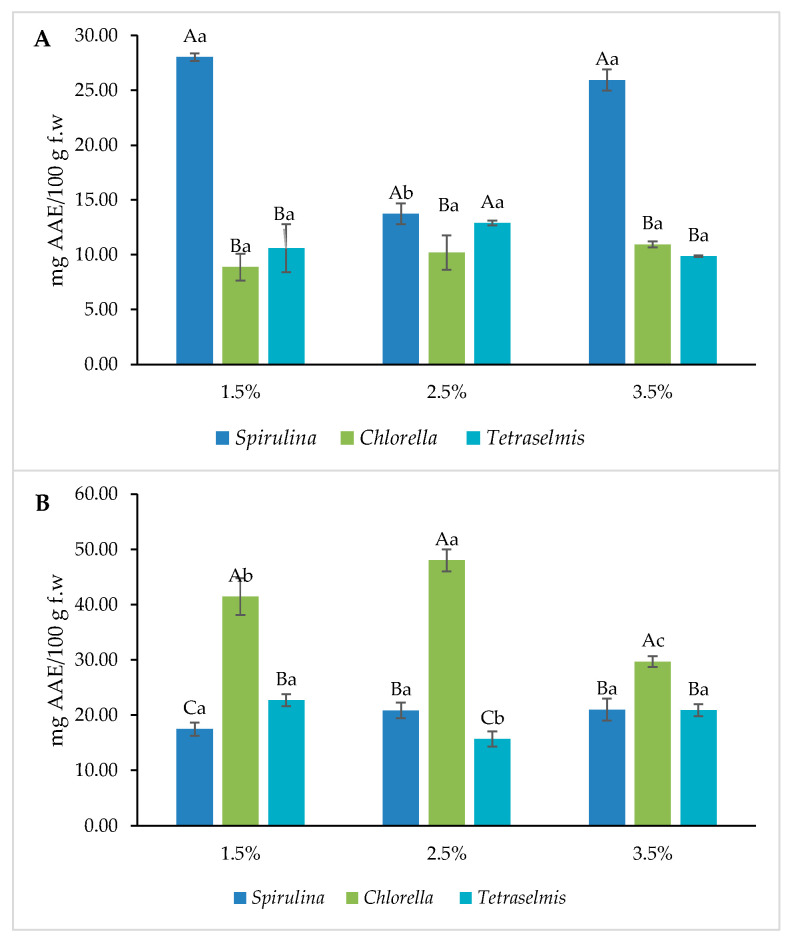
Ferric reducing antioxidant power (FRAP) for microalgae containing samples. (**A**): Crackers and (**B**): Grissini. Different capital letters indicate significant differences between formulations baked at different concentrations with the same microalgae. Different lowercase letters indicate significant differences between microalgae at the same concentration. The criterion for statistical significance was *p* < 0.05.

Nutrimental results in terms of total phenolic content (TPC) and ferric reducing antioxidant power (FRAP), are shown in Figure 4 and Figure 5. The crackers that included *Spirulina* showed the highest results in all the concentrations evaluated, with no significant differences between them. However, *Chlorella* obtained the highest yields in terms of grissini; as with the crackers, the three concentrations showed higher values than the other two microalgae evaluated. As previously described by Koller et al. [8], microalgae are a rich source of antioxidant compounds; these properties are attributed to their pigments. Similar results in TPC had been observed in diverse studies such as in Sukhikh et al. [23], with similar microalgae concentrations (1, 3 and 6%) with better results at higher concentrations. Hernandez-Lopez et al. [24] also produced results like these with *Spirulina* biomass added to bread with a different strength. 

In accordance with the results for phenols, it was seen that the antioxidant activity measured by FRAP has similar results, with Spirulina being the algae with the best performance in the three concentrations for crackers, while for grissini, Chlorella had better results in the three concentrations. Previous reports have demonstrated that microalgal biomass contains important amounts of polyphenols and carotenoids, contributing to its antioxidant capacity [25,26].

**Figure 6:**
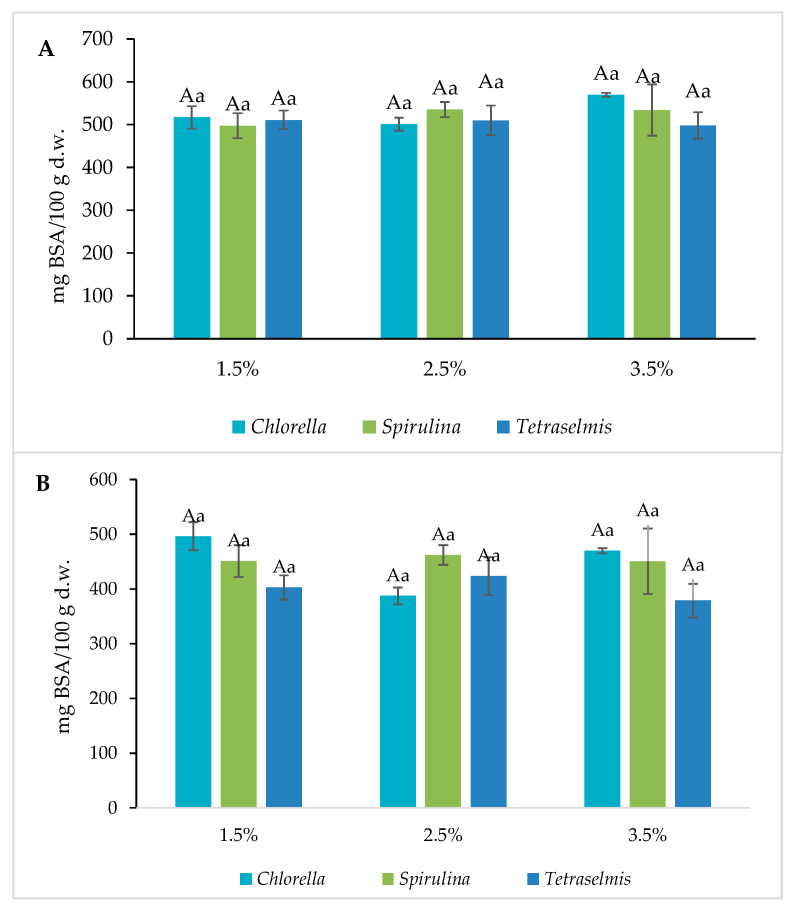
Total protein content for microalgae containing samples. (**A**): Grissini and (**B**): Crackers. Different capital letters indicate significant differences between formulations baked at different concentrations with the same microalgae. Different lowercase letters indicate significant differences between microalgae at the same concentration. The criterion for statistical significance was *p* < 0.05.

For both crackers and grissini, the values obtained for the total protein content (Figure 6) did not show significant differences with increasing concentrations. However, the results for crackers are slightly higher overall than for grissini.

The utilization of microalgae as a source of protein in foods is usually justified with the high content (40–60% dry matter) and high-quality protein in microalgae [27,28]. In particular, baked products are suitable for the incorporation of microalgae in order to obtain added-value food goods [29]. As for example, the consumption of Spirulina has been studied in athletes to evaluate if there is an enhancement in their performance due to Spirulina supplementation [30].

Contrary to our results, previous works reported the protein addition of microalgae in food. Also, Rodriguez DeMarco et al. [31] achieved an increase in total protein content with the addition of Spirulina to a pasta matrix. According to Montevecchi et al. [32], the incorporation of 1 and 2% of Spirulina in bread resulted in a higher protein content of the final products (3.17% and 5.12%, respectively). Batista et al. [33] also found that the addition of Chlorella in crackers increased protein content in the resultant functional product.

Neither cracker nor grissini enrichment with Chlorella or Spirulina resulted in a higher protein content. These microalgae have been selected for large-scale production and application in the food industry because of their acceptability and particularly significant protein content [27]. Wheat also presents protein in its composition; indeed, the protein in wheat is responsible for the bread’s structure. Results suggest that, while the replacement percentages of gradual microalgae addition did not modify the final total protein content of the evaluated products, it would be necessary to observe the amino acid profile in the reformulated products.

### 3.3. Sensory Evaluation

Sensory evaluation was carried out in terms of global acceptance, flavor, firmness, and crunchiness. Results are shown in Table 5 and Table 6.

In the case of crackers, the highest overall acceptance result is observed for both Chlorella 2.5% and 3.5% with an even 62% of evaluation above 7. Even though on the separate evaluation the highest flavor score is for the 1.5% Spirulina, in which 69% of the participants scored this sample with a value of 7 or higher. These results are not surprising, because according to what Niccolai et al. [34] documented in a sensory test of “crostini” with Spirulina, tasters tended to favor the formulations or samples with a smaller concentration of microalgae. As the number of microalgae in the samples increased, they became increasingly unpalatable to the participants. Also as reported by Batista et al. [33], in a sensory test of wheat crackers, samples containing Spirulina were better evaluated than those containing Tetraselmis or Chlorella.

With the grissini (Table 6), the samples with *Chlorella* stand out more in both flavor and global acceptance, being the 3.5% concentration the one with the higher overall acceptance score, with a 67% and a 69% evaluation score of 7 or higher respectively. In addition, comments were received that the samples with *Chlorella* offered a more appealing and appetizing appearance, as opposed to the samples with *Tetraselmis*, for example, which appeared “too dark” to the participants; this is consistent with the findings presented by Batista et al. [33]. In a previous work, the acceptance of the grissini “breadsticks” with the microalgae addition was already reported by Garcia-Segovia et al. [22] with good results.

*Tetraselmis* in general terms has been evaluated as very salty, and the appearance it confers to the samples is not pleasant, due to the fact that the biomass is very dark; similar results were observed by Chacon-Lee et al. [35]. According to the results obtained by Hernandez-Lopez et al. [17], foods containing microalgae are pleasant for consumers, and the marine aftertaste is not entirely unpleasant and could be considered a good option when eating seafood.

### 3.4. Sensory Acceptance Selection

Following the sensory evaluation, the products with the higher overall acceptance and flavor scores were chosen. These parameters were determinants in the decision over which products would be evaluated in vitro since products that were not well accepted by the panelists would not fit the parameters. These included crackers with a 1.5% addition of Spirulina, as well as grissini with a 3.5% addition of Chlorella. None of the samples containing Tetraselmis were selected, primarily due to their darker greenish coloration and an undesirable salty taste. According to the overall results, the selected products were deeply evaluated considering the quality of the protein by analyzing the EAA and subjecting the samples to an in vitro digestion, to assess the bioavailability of the bioactive compounds present in the baked goods; these results are discussed below.

### 3.5. Amino Acids Profile of Selected Products

Considering the selected products and the need to know the amino acids profile in the reformulated products, a free amino acid profile was assessed (Table 7).

According to Palmer, S [36], an adult’s total amino acids recommended daily allowance is around 84 mg/Kg. With this in mind and considering the low concentrations of microalgae used for this analysis, levels of free essential amino acids could be a potential and suitable source. Montevecchi et al. [32] used a Spirulina biomass to enrich baked bread with semi-whole flour. The obtained results in that study can be compared to the ones obtained in the present paper, even though the concentrations used by them were higher than the ones used here. In general terms, valine was the highest amino acid present both in Spirulina and Chlorella. These results are also in line with those reported by Terriente-Palacios and Castellari [37] in which they measured the amino acids content in crackers with Spirulina.

### 3.6. In Vitro Gastrointestinal Essay Evaluating TPC/AOX of Selected Products

Since the total antioxidant value of those selected products increased in relation to the control, an in vitro gastrointestinal essay was conducted to observe the bioavailability and potential health benefits of these enhanced antioxidant properties.

Although antioxidant values by FRAP methodology were found to be lower in the grissini matrix (10–30 mg EAA/100 g), in comparison with the results obtained for crackers (20–40 mg EAA/100 g), the bioavailability of these bioactives increased intensively after digestion (Table 8); grissini had an average increase of 64% and crackers an average increase of 93%. There was no clear correlation between the increase in bioavailability of antioxidant compounds after digestion with a higher content of microalgae. The increase in antioxidant activity and polyphenol content obtained after the in vitro bioavailability analysis is an estimate of what could be actually utilized during the digestion of in vivo subjects. Furthermore, the increase may be due to the fact that the enzymatic processes to which the food and the microalgae contained therein were subjected may have disrupted the cell, obtaining the components within the microalgae that were not extracted during cooking [38].

Total polyphenol content followed the same tendency as the antioxidant compounds, with greater values and bioavailability after the digestion in the cracker matrix. Grissini, in general, highlighted their low polyphenol content before and after digestion.

Although the digestibility and bioaccessibility of antioxidant and polyphenols differed deeply between algal species, in general, in vivo studies showed an enhancement in this bioactivity [39]. Previous research has already studied the bioavailability of the different antioxidant components of microalgae [40]. Phenolic compounds also showed this tendency of a bioavailability increment after digestion in micro-algae [41]. Digestion implies the degradation of structures and release of compound, such as polyphenols [42].

The incorporation of microalgae into baked products and the digestion effect in polyphenols and antioxidant activity have already been reported in cookies [33] and in baked goods [12].

## 4. Conclusions

In light of the growing popularity of plant-based foods due to their health benefits and lower environmental impact, microalgae emerge as a promising source of high-value compounds. Consistent with prior research into microalgae, particularly *Chlorella* and *Spirulina* prove to be rich sources of protein and essential amino acids. This study reinforces the theory that *Spirulina* and *Chlorella* exhibit high levels of antioxidant activity and phenolic content in baked products. In the end, the products showed a higher nutritional value when compared to the control samples without microalgae. Although “new” spices such as *Tetraselmis* are being evaluated for consumption and commercialization, the inherent green color and distinct salty taste pose challenges. Nevertheless, it has been demonstrated that, in low concentrations, this taste can be masked depending on the matrices used, and the green color can even enhance the product’s appeal. While regular consumers may be accustomed to these characteristics, broadening consumption requires addressing these limitations. Therefore, ongoing efforts are necessary to find innovative solutions that cater to both the nutritional content and the product’s appearance, ensuring a broader appeal and acceptance among non-regular consumers. Also, the potential of these microalgae to properly develop a functional food is a promising way to continue research in this matter.

## 5. Limitations

The future of this edible microalgae production is related to the development of large-scale photobioreactors, where environmental conditions can be managed and all safety criteria assured [6]. Economic and environmental difficulties in nutrient and bioactive or interesting compounds extraction process also limit the utilization of microalgae by the food industry. With regard to consumers, the microalgae suffer sensory acceptance challenges related to their peculiar flavor that can be overcome with food technology [7]. Furthermore, even if microalgae are shown to contain antioxidant compounds and this activity can be measured in food, it is still only a potential opportunity for consumers to really take advantage of this activity. This is because there is no certainty about the necessary amount of antioxidant activity in the body. More studies should be carried out in this area to better understand the utilization of these compounds in the human daily diet. 

## Figures and Tables

**Table 1:** Crackers Formulation.

Samples	Flour Type 55 (g)	Baking Powder (g)	Cold Water (mL)	Yeast (g)	Salt (g)	Microalgae (g)
**CK**	156.2	1.6	94.0	4.7	3.1	-
**1.5%**	153.8	1.6	94.0	4.7	3.1	2.3
**2.5%**	152.3	1.6	94.0	4.7	3.1	3.9
**3.5%**	150.7	1.6	94.0	4.7	3.1	5.5

CK: Control samples are referred to samples with no microalgae in the formulation.

**Table 2:** Grissini Formulation.

Samples	Flour Type 55 (g)	Baking Powder (g)	Cold Water (mL)	Olive Oil (mL)	Yeast (g)	Salt (g)	Microalgae (g)
**CK**	416.6	4.2	208.3	41.6	12.5	8.3	-
**1.50%**	410.4	4.2	208.3	41.6	12.5	8.3	6.3
**2.50%**	406.2	4.2	208.3	41.6	12.5	8.3	10.4
**3.50%**	404.2	4.2	208.3	41.6	12.5	8.3	12.5

CK: Control samples are referred to samples with no microalgae in the formulation.

**Table 3:** Chromatographic gradient.

Time (min)	Flow (mL/min)	Mobile Phase A (%)	Mobile Phase B (%)
0.00	0.40	99.0	1.0
2.00	0.40	99.0	1.0
8.00	0.40	30.0	70.0
10.00	0.40	99.0	1.0
13.00	0.40	99.0	1.0

**Table 4:** Color recording for the baked goods samples including all the microalgae concentrations.

Color Recordings							
Grissini	L*	a*	b*	Crackers	L*	a*	b*
CK	65.29	12.51	38.20	CK	65.02	12.14	20.91
Ch. 1.5%	57.85 ± 0.51 Aa	0.97 ± 0.88 Ab	38.30 ± 1.10 Aa	Ch. 1.5%	64.92 ± 3.60 Aa	5.70 ± 2.54 Aa	22.81 ± 0.64 Aa
Ch. 2.5%	56.72 ± 3.89 Aa	−1.56 ± 0.41 Bc	32.29 ± 1.39 Ab	Ch. 2.5%	63.54 ± 3.77 Aa	6.01 ± 2.21 Aa	16.59 ± 2.45 Ab
Ch. 3.5%	44.45 ± 3.52 Bb	5.83. ± 1.04 Aa	24.47 ± 1.72 Aa	Ch. 3.5%	63.46 ± 1.87 Aa	0.83 ± 3.16 Ab	19.46 ± 1.83 Ab
Sp. 1.5%	54.58 ± 2.11 Bb	1.92 ± 0.74 Ac	28.08 ± 1.40 Ba	Sp. 1.5%	66.24 ± 2.15 Aa	1.52 ± 1.27 Ba	9.08 ± 1.37 Ca
Sp. 2.5%	57.40 ± 1.70 Ab	0.14 ± 0.19 Ab	18.81 ± 1.30 Bc	Sp. 2.5%	60.98 ± 1.49 Ba	1.248 ± 1.40 Ba	10.05 ± 1.40 Ba
Sp. 3.5%	66.02 ± 5.42 Aa	4.48 ± 1.41 Aa	20.27 ± 2.30 Bb	Sp. 3.5%	67.40 ± 4.12 Aa	1.356 ± 0.60 Aa	7.37 ± 2.04 Ca
Ts. 1.5%	40.44 ± 1.23 Ca	−2.77 ± 2.37 Bb	28.18 ± 1.50 Cb	Ts. 1.5%	60.05 ± 1.16 Ab	−7.00 ± 2.37 Ca	16.81 ± 0.55 Ba
Ts. 2.5%	37.21 ± 1.54 Bb	−11.12 ± 0.92 Cc	35.62 ± 0.99 Bb	Ts. 2.5%	59.45 ± 1.28 Aab	−8.14 ± 2.56 Ca	16.28 ± 1.13 Aab
Ts. 3.5%	46.38 ± 4.02 Bb	0.16 ± 0.45 Ba	21.18 ± 1.29 Ba	Ts. 3.5%	56.43 ± 2.92 Bb	−8.65 ± 1.43 Ba	14.40 ± 1.56 Bb

Samples Abbreviations: Ch: *Chlorella*, Sp. *Spirulina* and Ts. *Tetraselmis*. Different percentages indicate the amount of flour substituted. Different capital letters indicate significant differences between formulations baked with different concentrations with the same microalgae. Different lowercase letters indicate significant differences between microalgae at the same concentration. The criterion for statistical significance was *p* < 0.05.

**Table 5:** Crackers evaluation percentages with results above 7 on a 9-point hedonic scale.

Crackers		Crunchiness	Firmness	Flavor	Global Acceptance
*Chlorella*	1.50%	79%	72%	59%	55%
	2.50%	72%	68%	52%	62%
	3.50%	83%	72%	48%	62%
*Spirulina*	1.50%	83%	86%	69%	62%
	2.50%	83%	72%	41%	57%
	3.50%	97%	90%	48%	55%
*Tetraselmis*	1.50%	83%	93%	38%	31%
	2.50%	90%	83%	38%	38%
	3.50%	79%	76%	31%	28%

Sensory evaluation for crackers samples containing *Chlorella*, *Spirulina* and *Tetraselmis* at different concentrations (1.5%, 2.5% and 3.5%). Scores were assessed using a 9-point hedonic scale (from 1: dislike extremely to 9: like extremely).

**Table 6:** Grissini evaluation percentages with results above 7 on a 9-point hedonic scale.

Grissini		Crunchiness	Firmness	Flavor	Global Acceptance
*Chlorella*	1.50%	54%	56%	67%	59%
	2.50%	44%	51%	70%	64%
	3.50%	64%	74%	69%	67%
*Spirulina*	1.50%	40%	49%	54%	51%
	2.50%	29%	37%	43%	49%
	3.50%	60%	68%	63%	49%
*Tetraselmis*	1.50%	51%	54%	32%	32%
	2.50%	40%	59%	30%	38%
	3.50%	54%	62%	41%	38%

Sensory evaluation for grissini samples containing *Chlorella*, *Spirulina* and *Tetraselmis* at different concentrations (1.5%, 2.5% and 3.5%). Scores were assessed using a 9-point hedonic scale (from 1: dislike extremely to 9: like extremely).

**Table 7:** Essential free amino acids present in baked goods samples.

Essential Amino Acids mg aa/100 g Total Product		
	Crackers Spirulina 1.5%	Grissini Chlorella 3.5%
Isoleucine	11.6	4.0
Leucine	16.5	5.1
Lysine	0.1	0.2
Threonine	-	0.2
Tryptophan	11.6	6.8
Valine	27.8	27.1

**Table 8:** Comparison of TPC and AOX content before and after a bioaccessibility assay.

TPC & AOX Activity before and after Digestion			
TPC	Samples	Pre-Digestion	Post Digestion
mg Eq GA/100 g f.w.	*Crackers Spirulina 1.5%*	35.17 ± 3.34	317.1 ± 22.4
	*Grissini Chlorella 3.5%*	16.4 ± 0.5	114.9 ± 0.7
FRAP	Samples	Pre-Digestion	Post Digestion
mg Eq AA/100 g f.w.	*Crackers Spirulina 1.5%*	17.4 ± 1.1	182.8 ± 7.9
	*Grissini Chlorella 3.5%*	10.9 ± 0.2	96.5 ± 2.9

## Data Availability

Data is contained within this article.

## References

[1] Ren Y., Sun H., Deng J., Huang J., Chen F. (2021). Carotenoid Production from Microalgae: Biosynthesis, Salinity Responses and Novel Biotechnologies. Mar. Drugs.

[2] Geyik O., Hadjikakou M., Bryan B.A. (2020). Spatiotemporal Trends in Adequacy of Dietary Nutrient Production and Food Sources. Glob. Food Sec..

[3] Dineshbabu G., Goswami G., Kumar R., Sinha A., Das D. (2019). Microalgae–Nutritious, Sustainable Aqua- and Animal Feed Source. J. Funct. Foods.

[4] García J.L., de Vicente M., Galán B. (2017). Microalgae, Old Sustainable Food and Fashion Nutraceuticals. Microb. Biotechnol..

[5] Gohara-Beirigo A.K., Matsudo M.C., Cezare-Gomes E.A., de Carvalho J.C.M., Danesi E.D.G. (2022). Microalgae Trends toward Functional Staple Food Incorporation: Sustainable Alternative for Human Health Improvement. Trends Food Sci. Technol..

[6] Matos Â.P. (2017). The Impact of Microalgae in Food Science and Technology. JAOCS J. Am. Oil Chem. Soc..

[7] Tibbetts S.M., Milley J.E., Lall S.P. (2015). Chemical Composition and Nutritional Properties of Freshwater and Marine Microalgal Biomass Cultured in Photobioreactors. J. Appl. Phycol..

[8] Koller M., Muhr A., Braunegg G. (2014). Microalgae as Versatile Cellular Factories for Valued Products. Algal Res..

[9] Watanabe F., Takenaka S., Kittaka-Katsura H., Ebara S., Miyamoto E. (2002). Characterization and Bioavailability of Vitamin B12-Compounds from Edible Algae. J. Nutr. Sci. Vitaminol..

[10] Verni M., Demarinis C., Rizzello C.G., Pontonio E. (2023). Bioprocessing to Preserve and Improve Microalgae Nutritional and Functional Potential: Novel Insight and Perspectives. Foods.

[11] Moon S.-H., Cho S.-J. (2023). Evaluation of the Antioxidant Activity of Tetraselmis Chuii after in Vitro Gastrointestinal Digestion and Investigation of Its Antioxidant Peptides. Algal Res..

[12] Lafarga T., Mayre E., Echeverria G., Viñas I., Villaró S., Acién-Fernández F.G., Castellari M., Aguiló-Aguayo I. (2019). Potential of the Microalgae Nannochloropsis and Tetraselmis for Being Used as Innovative Ingredients in Baked Goods. LWT.

[13] Lafarga T., Villaró S., Bobo G., Simó J., Aguiló-Aguayo I. (2019). Bioaccessibility and Antioxidant Activity of Phenolic Compounds in Cooked Pulses. Int. J. Food Sci. Technol..

[14] Nicolau-Lapeña I., Abadias M., Bobo G., Lafarga T., Viñas I., Aguiló-Aguayo I. (2021). Antioxidant and Antimicrobial Activities of Ginseng Extract, Ferulic Acid, and Noni Juice: Evaluation of Their Potential to Be Incorporated in Food. J. Food Process Preserv..

[15] Lowry O.H., Rosebrough N.J., Farr A.L., Randall R.J. (1951). Protein measurement with the folin phenol reagent. J. Biol. Chem..

[16] Millar K.A., Barry-Ryan C., Burke R., Hussey K., McCarthy S., Gallagher E. (2017). Effect of Pulse Flours on the Physiochemical Characteristics and Sensory Acceptance of Baked Crackers. Int. J. Food Sci. Technol..

[17] Hernández-López I., Benavente Valdés J.R., Castellari M., Aguiló-Aguayo I., Morillas-España A., Sánchez-Zurano A., Acién-Fernández F.G., Lafarga T. (2021). Utilisation of the Marine Microalgae *Nannochloropsis* Sp. and *Tetraselmis* Sp. as Innovative Ingredients in the Formulation of Wheat Tortillas. Algal Res..

[18] Kıvrak İ., Kıvrak Ş., Harmandar M. (2014). Free Amino Acid Profiling in the Giant Puffball Mushroom (Calvatia Gigantea) Using UPLC-MS/MS. Food Chem..

[19] Lafarga T., Acién-Fernández F.G., Castellari M., Villaró S., Bobo G., Aguiló-Aguayo I. (2019). Effect of Microalgae Incorporation on the Physicochemical, Nutritional, and Sensorial Properties of an Innovative Broccoli Soup. LWT.

[20] Batista A.P., Niccolai A., Bursic I., Sousa I., Raymundo A., Rodolfi L., Biondi N., Tredici M.R. (2019). Microalgae as Functional Ingredients in Savory Food Products: Application to Wheat Crackers. Foods.

[21] da Silva Figueira F., de Moraes Crizel T., Rubira Silva C., de las Mercedes Salas-Mellado M. (2011). Pão Sem Glúten Enriquecido Com a Microalga Spirulina Platensis. Braz. J. Food Technol..

[22] García-Segovia P., García Alcaraz V., Tárrega A., Martínez-Monzó J. (2020). Consumer Perception and Acceptability of Microalgae Based Breadstick. Food Sci. Technol. Int..

[23] Sukhikh S., Ivanova S., Dolganyuk V., Pilevinova I., Prosekov A., Ulrikh E., Noskova S., Michaud P., Babich O. (2022). Evaluation of the Prospects for the Use of Microalgae in Functional Bread Production. Appl. Sci..

[24] Hernández-López I., Alamprese C., Cappa C., Prieto-Santiago V., Abadias M., Aguiló-Aguayo I. (2023). Effect of Spirulina in Bread Formulated with Wheat Flours of Different Alveograph Strength. Foods.

[25] Goiris K., Muylaert K., Fraeye I., Foubert I., De Brabanter J., De Cooman L. (2012). Antioxidant Potential of Microalgae in Relation to Their Phenolic and Carotenoid Content. J. Appl. Phycol..

[26] Custódio L., Justo T., Silvestre L., Barradas A., Duarte C.V., Pereira H., Barreira L., Rauter A.P., Alberício F., Varela J. (2012). Microalgae of Different Phyla Display Antioxidant, Metal Chelating and Acetylcholinesterase Inhibitory Activities. Food Chem..

[27] Becker E.W. (2007). Micro-Algae as a Source of Protein. Biotechnol. Adv..

[28] Wang Y., Tibbetts S.M., McGinn P.J. (2021). Microalgae as Sources of High-Quality Protein for Human Food and Protein Supplements. Foods.

[29] Raymundo A., Fradinho P., Nunes M.C. (2023). Application of Microalgae in Baked Goods and Pasta. Handbook of Food and Feed from Microalgae: Production, Application, Regulation, and Sustainability.

[30] Calella P., Cerullo G., Di Dio M., Liguori F., Di Onofrio V., Gallè F., Liguori G. (2022). Antioxidant, Anti-Inflammatory and Immunomodulatory Effects of Spirulina in Exercise and Sport: A Systematic Review. Front. Nutr..

[31] Rodríguez De Marco E., Steffolani M.E., Martínez C.S., León A.E. (2014). Effects of Spirulina Biomass on the Technological and Nutritional Quality of Bread Wheat Pasta. LWT.

[32] Montevecchi G., Santunione G., Licciardello F., Köker Ö., Masino F., Antonelli A. (2022). Enrichment of Wheat Flour with Spirulina. Evaluation of Thermal Damage to Essential Amino Acids during Bread Preparation. Food Res. Int..

[33] Batista A.P., Niccolai A., Fradinho P., Fragoso S., Bursic I., Rodolfi L., Biondi N., Tredici M.R., Sousa I., Raymundo A. (2017). Microalgae Biomass as an Alternative Ingredient in Cookies: Sensory, Physical and Chemical Properties, Antioxidant Activity and in Vitro Digestibility. Algal Res..

[34] Niccolai A., Venturi M., Galli V., Pini N., Rodolfi L., Biondi N., D’Ottavio M., Batista A.P., Raymundo A., Granchi L. (2019). Development of New Microalgae-Based Sourdough “Crostini”: Functional Effects of Arthrospira Platensis (Spirulina) Addition. Sci. Rep..

[35] Chacón-Lee T.L., González-Mariño G.E. (2010). Microalgae for “Healthy” Foods-Possibilities and Challenges. Compr. Rev. Food Sci. Food Saf..

[36] Palmer S. (1990). Recommended Dietary Allowances, Tenth Edition.

[37] Terriente-Palacios C., Castellari M. (2022). Levels of Taurine, Hypotaurine and Homotaurine, and Amino Acids Profiles in Selected Commercial Seaweeds, Microalgae, and Algae-Enriched Food Products. Food Chem..

[38] Thakur N., Raigond P., Singh Y., Mishra T., Singh B., Lal M.K., Dutt S. (2020). Recent Updates on Bioaccessibility of Phytonutrients. Trends Food Sci. Technol..

[39] Demarco M., Oliveira de Moraes J., Matos Â.P., Derner R.B., de Farias Neves F., Tribuzi G. (2022). Digestibility, Bioaccessibility and Bioactivity of Compounds from Algae. Trends Food Sci. Technol..

[40] Ranga Rao A., Baskaran V., Sarada R., Ravishankar G.A. (2013). In Vivo Bioavailability and Antioxidant Activity of Carotenoids from Microalgal Biomass—A Repeated Dose Study. Food Res. Int..

[41] Muszyńska B., Krakowska A., Lazur J., Jękot B., Zimmer Ł., Szewczyk A., Sułkowska-Ziaja K., Poleszak E., Opoka W. (2018). Bioaccessibility of Phenolic Compounds, Lutein, and Bioelements of Preparations Containing Chlorella Vulgaris in Artificial Digestive Juices. J. Appl. Phycol..

[42] Wojtunik-Kulesza K., Oniszczuk A., Oniszczuk T., Combrzyński M., Nowakowska D., Matwijczuk A. (2020). Influence of in Vitro Digestion on Composition, Bioaccessibility and Antioxidant Activity of Food Polyphenols—A Non-Systematic Review. Nutrients.

